# Managing Perforated Diverticulitis: An Overview of Treatment Trends and Clinical Outcomes at a Single Centre in the United Kingdom

**DOI:** 10.7759/cureus.72591

**Published:** 2024-10-28

**Authors:** Kush Patel, Ahmad Shehadeh, Kyrllos Farag, Vladimir Nichita, Ahmed Esawi, Rishi Sen, Elisabeth Drye, Sanad Isswiasi

**Affiliations:** 1 General Surgery, West Suffolk NHS Foundation Trust, Bury St Edmunds, GBR; 2 General and Colorectal Surgery, Barnsley Hospital NHS Foundation Trust, Barnsley, GBR; 3 General Surgery and Oncoplastic Breast Surgery, West Suffolk Hospital NHS Trust, Bury St Edmunds, GBR; 4 General Surgery, Peterborough City Hospital, Peterborough, GBR; 5 Surgery, West Suffolk Hospital, Bury St Edmunds, GBR; 6 General Surgery, West Suffolk Hospital, Bury St Edmunds, GBR

**Keywords:** colostomy closure, colostomy reversal, complicated diverticulitis, perforated colon, perforated diverticulitis, purulent peritonitis

## Abstract

Introduction

Perforation represents the most critical manifestation of complicated diverticulitis. In 2008, it was estimated that about 2,000 cases of perforated diverticulitis (PD) were diagnosed in the United Kingdom (UK). Management of PD is evolving with considerable variation in approaches between hospitals and countries with an increased trend towards a conservative approach.

Objective

Our aim is to provide a comprehensive overview of the management strategies and treatment outcomes for PD, with a particular focus on the influence of abscess size and the presence of distant air (DA) on the success of conservative management.

Methods

Data from 112 patients admitted with PD to a single district hospital in the UK between 2013 and 2018 were retrospectively analysed. CT scan reports and images were examined to assess the size and number of abscesses, as well as the presence of DA. Failed initial management was defined as the need for an alternative therapeutic option after 48 hours during the index admission or readmission within 12 weeks. Follow-up data were also reviewed to evaluate the need for elective resection and stoma reversal.

Result

In this cohort of 112 patients with PD, a variety of treatment strategies were employed. Antibiotic therapy alone was successful in 46 patients (41%). Radiological management was successful in only six patients (5%). Surgical washout was required in 12 cases (11%), while resection was performed in 40 cases (36%). Best supportive care was provided to eight patients (7%) who were considered unfit for invasive interventions. The success rate was higher in cases with smaller abscesses and no DA (p <0.05). Specifically, 30 out of 45 patients (66.6%) with abscesses less than 4 cm and no DA were managed successfully with conservative treatment, whereas the success rate dropped to 14 out of 30 patients (47%) when DA was present (p<0.05). For abscesses larger than 4 cm, the success rate was seven out of 20 patients (35%) without DA and significantly lower at two out of 20 patients (10%) with DA (p=0.01). The data also show a shift towards increased conservative management over the six-year period, with a steady reduction in the number of surgical interventions. However, 12 patients (19%) were readmitted with complicated diverticulitis after the initial non-resectional management.

Conclusion

We observed a shift towards more conservative, non-operative management of acute complicated diverticulitis with perforation over the six-year period, likely influenced by advancements in diagnostic and interventional radiology, antibiotic therapy, and minimally invasive techniques. Our data also stress that cases of PD with distant extraluminal air or larger abscesses are less suited to conservative treatment, often necessitating traditional surgical interventions. Long-term follow-up showed a moderate rate of readmissions after non-resectional management, and while stoma reversal was successful in a proportion of patients, many either opted to live with the stoma or were deemed unsuitable for reversal. A larger, multicentre prospective study would likely provide more robust data on this subject.

## Introduction

Perforation represents the most critical manifestation of complicated diverticulitis, accounting for 75% of diverticulitis-related surgical emergencies [1,2]. The management of acute complicated diverticulitis has undergone significant changes due to advancements in surgical techniques and supportive medical care [3,4]. In the past, complicated diverticulitis involving perforation or abscess formation was typically addressed through surgery, often resulting in bowel resection and end colostomy. It is noteworthy that a considerable number of patients (35%-60%) who underwent surgery ended up with permanent stomas, in addition to facing associated risks and complications [5-8].

Currently, the focus is on transforming urgent surgical cases into potential elective care scenarios. This shift has been facilitated by the introduction of more precise scanning methods, newer antibiotics, and enhanced radiology-guided drainage techniques [9-11]. Research has demonstrated favourable outcomes for the non-operative management of patients with perforated diverticulitis (PD) accompanied by extraluminal air [12-14]. Consequently, surgical intervention is now reserved for cases where aggressive non-operative treatment methods have been exhausted or where the patient is unsuitable for non-surgical options. Furthermore, published reports highlight the effectiveness of laparoscopic non-resectional procedures, such as laparoscopic peritoneal lavage and suture closure of perforation, as alternative approaches to emergency surgery, allowing for elective surgical resection if necessary [15-17]. However, there is limited data on the management of PD, particularly in the United Kingdom (UK), where studies have been relatively sparse. This gap highlights the need for more research to better understand trends in treatment outcomes.

Our objective is to present a thorough overview of the management approaches and treatment outcomes for PD), with particular emphasis on how abscess size and the presence of distant air (DA) impact the management of PD.

Part of these data were previously presented as a meeting abstract and a poster at the Annual Congress of the Association of Surgeons of Great Britain and Ireland [18], held from 4th to 8th May 2021.

## Materials and methods

We conducted a retrospective review of all patients admitted with PD between January 2013 and December 2018 at Peterborough City Hospital, part of the North West Anglia NHS Foundation Trust, UK. Patients were identified using ICD-10 code - K57.2 (diverticular disease of the large intestine with perforation and abscess), based on the World Health Organization’s classification [19].

Data on patient demographics (age, gender), length of stay, previous admissions, radiology reports, treatment modalities, and outcomes were extracted from the Sunquest ICE database (Sunquest Information Systems Inc., Tucson, AZ), as well as from the hospital's online medical records and correspondence.

At our centre, the conservative management of perforated diverticulitis involves close monitoring by a multidisciplinary team, including surgeons, microbiologists, and dietitians. The standard regimen typically includes at least one week of intravenous piperacillin-tazobactam (4.5 g every eight hours). For patients allergic to penicillin, we administer a combination of intravenous ciprofloxacin (400 mg every 12 hours) and metronidazole (500 mg every eight hours) for at least a week, supplemented with intravenous gentamicin (5-7 mg/kg lean body weight once daily, with subsequent doses adjusted according to serum-gentamicin concentration) for the first three days. Upon clinical improvement, we transition to oral therapy. Non-penicillin-allergic patients usually receive co-amoxiclav for at least a week, while penicillin-allergic patients are given an oral combination of ciprofloxacin and metronidazole for the same duration. All in-patients with complicated diverticulitis are advised to follow a low-residue diet if oral intake is permitted. In severe cases, especially for those in intensive care, total parenteral nutrition may be required. The duration of therapy is tailored to the severity of the infection and the patient’s clinical response. Additionally, antibiotic adjustments may be guided by culture results and the clinical picture.

Failed initial management was defined as the need for an alternative therapeutic option after 48 hours during the index admission or readmission within 12 weeks. The size and number of diverticular abscesses were extracted from computed tomography (CT) scans. DA was defined as extraluminal air located more than 5 cm from the area of inflammation or abscess.

Statistical analyses were conducted using the Statistical Product and Service Solutions (SPSS, version 21.0; IBM SPSS Statistics for Windows, Armonk, NY) software. The statistical significance of the impact of abscess size on treatment outcomes was assessed using Fisher's exact test, with a P-value of < 0.05 considered statistically significant. Ethical approval was obtained from the Quality Governance and Compliance Department at North West Anglia NHS Foundation Trust (approval number: 2934).

## Results

Demographics and clinical characteristics

We initially included 115 patients; however, three patients (2.6%) were excluded as the perforation was secondary to colorectal malignancy. Two of these cases were identified through histological examination following emergency operations, while one was diagnosed during a follow-up colonoscopy and subsequently resected electively. Hence, the final cohort consisted of 112 patients, with a median age of 64 years (range: 36-99). Of these, 61 patients (54%) were female. The median BMI of the cohort was 28, and 40 patients (35%) were aged 55 years or younger. The median ASA score was 2. Notably, 27 patients (24%) had a history of previous diverticulitis, with five patients having had prior admissions. DA was present in 44 patients (39%). The median length of hospital stay (LOS) was 10 days. Table 1 summarises the demographics and clinical characteristics of the cohort.

**Table 1:** Demographic and clinical characteristics of patients with perforated diverticulitis Data represented as n (total=112), (%), and median with range (range) were mentioned.

Characteristics	Value
Median Age (Range)	64 (36-99)
Female - n (%) : Male - n (%)	61 (54%) : 51 (46%)
Median BMI (Range)	28 (18-57)
≤55-Year-Old - n (%)	40 (35%)
Median ASA (Range)	2 (1-4)
Previous Diverticulitis - n (%)	27 (24%)
Previous Admission - n (%)	5 (4.5%)
Median LOS in Days (Range)	10 (0-97)

Management approaches

**Figure 1:**
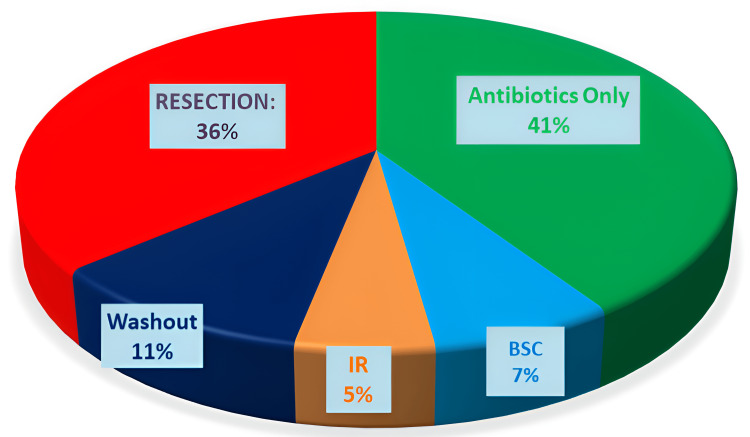
Overview of treatment approaches for perforated diverticulitis IR = interventional radiology, BSC = best supportive care

In this cohort, various treatment approaches were utilised. Antibiotic only treatment was successful in 46 patients (41%), while the best supportive care was provided to eight patients (7%) who were not medically fit for invasive interventions. Interventional radiological management (IR) was successful in six patients (5%), while surgical washout only was performed in 12 cases (11%). Resection was undertaken in 40 cases (36%). Among those who underwent resection, 36 patients (90%) received a colostomy (end colostomy), two patients (5%) had an end ileostomy, and two patients (5%) did not require a stoma. Figure 1 provides an overview of the therapeutic approaches and outcomes for PD in this cohort.

Abscess size and presence of distant air

**Figure 2:**
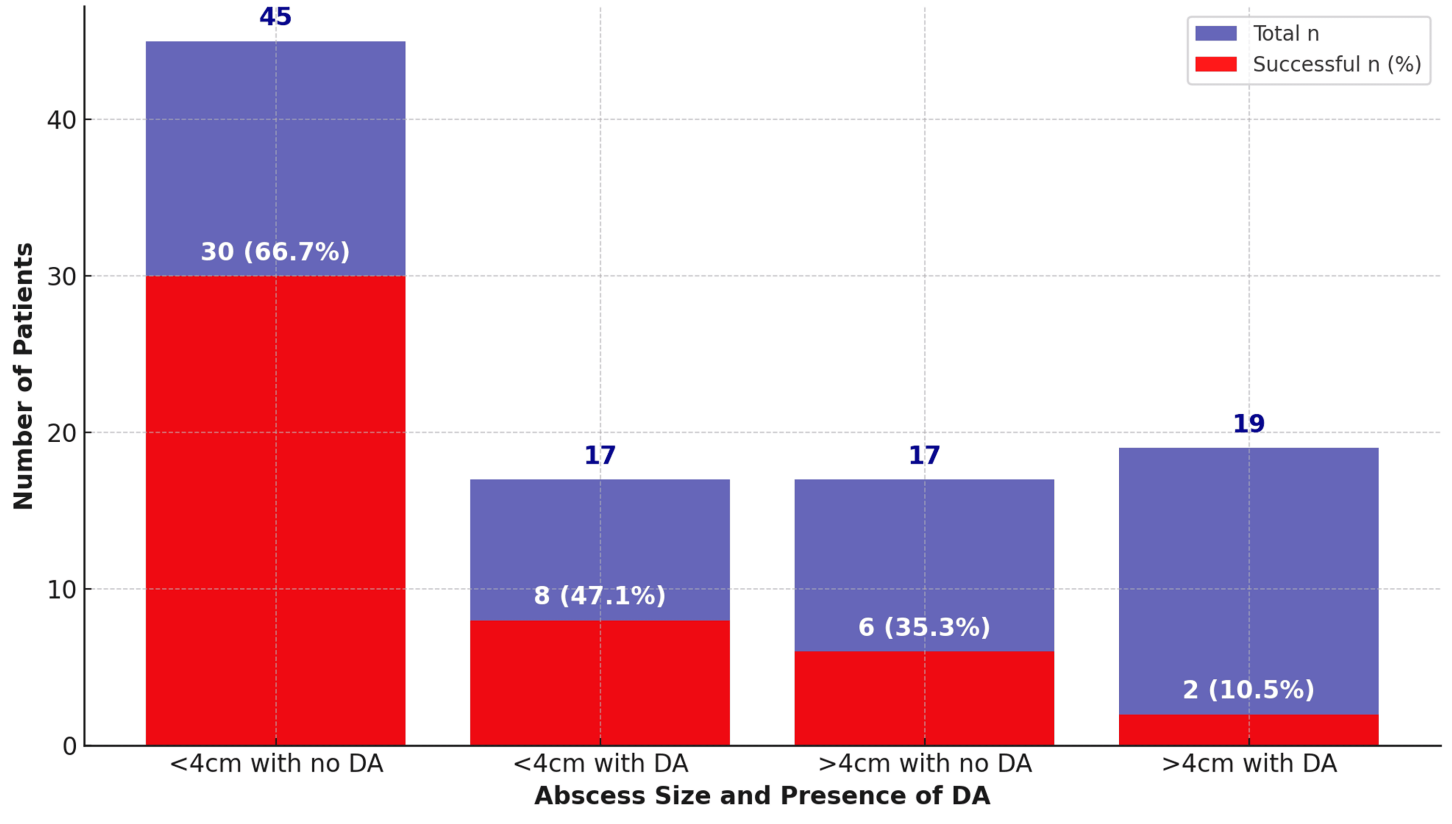
Impact of abscess size and presence of distant air (DA) on the success of conservative management

On further analysis of conservative management with antibiotics, the success rate was significantly higher in cases with smaller abscesses and no DA (p<0.05). Specifically, 30 out of 45 patients (66.6%) with abscesses less than 4 cm and no DA were managed successfully with conservative treatment, whereas the success rate dropped to 14 out of 30 patients (47%) when DA was present (p<0.05). For abscesses larger than 4 cm, the success rate was seven out of 20 patients (35%) without DA and significantly lower at two out of 20 patients (10%) with DA (p<0.05). Regardless of abscess size and the presence of DA, the success rate of conservative management in patients with more than one abscess was notably low, with only six out of 23 patients (26%) achieving successful outcomes (p<0.05). Figure 2 illustrates the impact of abscess size and the presence of DA on the success of conservative management.

Trends in treatment over time

**Figure 3:**
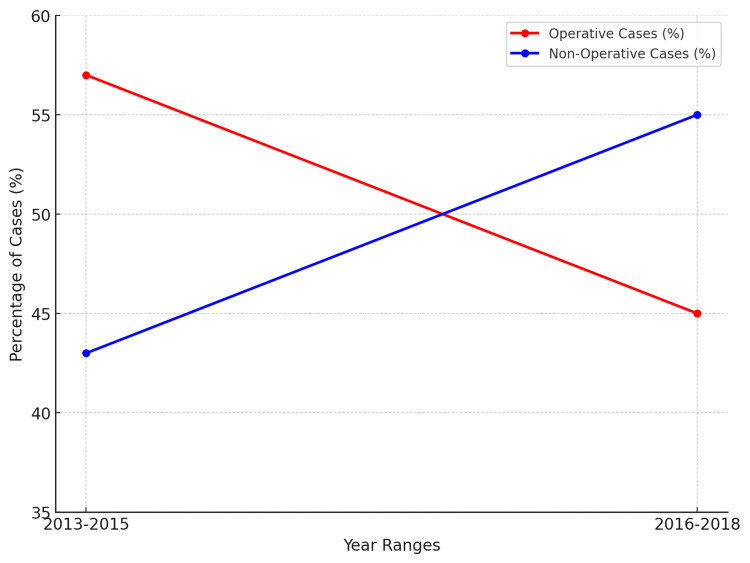
The trend in operative vs non-operative management of PD

There was a noticeable shift towards non-operative management (e.g. antibiotics with/without interventional radiology) over the study period. Between 2013 and 2015, 18 cases (43%) were managed non-operatively. However, between 2016 and 2018, non-operative management increased to 34 cases (55%). This is shown in Figure 3. Nevertheless, this shift was not statistically significant (p>0.05).

30-Day mortality and long-term outcomes

Nine patients (8%) died within 30 days of admission. Of these, seven patients (6%) passed away as expected following best supportive care, while two patients (2%) died after undergoing surgical washout procedures.

Over a median follow-up period of 42 months, 62 patients (55%) who were discharged after non-resectional management were tracked. Of these, 32 patients (50%) attended follow-up clinic appointments, and 17 patients (27%) remained asymptomatic. Fifteen patients (24%) were considered for elective resection, but only three patients (5%) ultimately underwent surgery. During the follow-up period, 12 patients (19%) were readmitted with complicated diverticulitis. Among them, four patients (33.3) had initially been treated with laparoscopic lavage, one (8.3%) had undergone radiological drainage, and the remaining seven (58%) were treated with antibiotics only. Notably, three patients (27%) who had initially been treated with laparoscopic lavage later required resection. The follow up findings are summarised in Table 2.

**Table 2:** Follow-up outcomes for patients discharged after non-resectional management of perforated diverticulitis

Category	Number (%)
Patients discharged after non-resectional management	62 (100%)
Patients attending follow-up clinic appointments	32 (50%)
Asymptomatic during follow-up	17 (27%)
Emergency readmission	12 (19%)
Assessed for elective resection:	15 (24%)
-Decision not to operate	12 (19%)
-Underwent elective surgery	3 (5%)

Of the 40 patients (36%) who were offered resectional surgery during the index acute admission, 38 (95%) were considered for reversal, but only 16 (40%) reversals were performed. Thirteen patients (32.5%) opted to live with the stoma, while nine patients (22.5%) were deemed high-risk candidates for reversal due to factors such as general fitness, advanced age, or high body weight.

## Discussion

The management of acute complicated diverticulitis has evolved significantly due to advancements in surgical techniques and improved medical care [3,4]. Historically, cases involving perforation or abscess formation were primarily treated surgically, often necessitating bowel resection and the creation of an end colostomy, both of which carried substantial risks of complications and mortality [20,21]. However, our study observed a marked shift towards more conservative management approaches over time, likely reflecting advancements in imaging technologies, the effectiveness of newer antibiotics, and the growing use of radiology-guided drainage techniques [9-11].

In our cohort, surgical resection was required in 40 cases (36%), while the majority of patients were successfully managed with less invasive options. The success rate for treating abscesses was the highest (n=30/45, 66.6%) for smaller abscesses (<4 cm) without DA. However, the success rate significantly decreased as abscess size increased or when DA was present, with larger abscesses (>4 cm) accompanied by DA having the lowest success rate (n=2/19, 10%). These findings are consistent with previous studies, which have similarly demonstrated the influence of abscess size and DA on treatment outcomes [10,12-14].

Peritoneal lavage has been shown to be a safe and effective treatment in several studies, demonstrating reduced morbidity and mortality compared to traditional surgical approaches [15-17]. In our cohort, surgical washout was initially successful in 12 cases (11%), with only four readmissions (33.3%) during the follow-up period, of which three patients (25%) required resection. Notably, the two post-operative deaths in our study occurred in patients who had undergone surgical washout.

Our data also reports that 12 patients (19%) were readmitted with complicated diverticulitis following initial non-resectional management. Previous studies have reported readmission rates ranging from 9.6% to 19% following non-surgical management [22-24].

In our centre, surgeons reversed 16 stomas (40%), and there were all end colostomies. Thirteen patients (32.5%) opted to live with the stoma, while nine patients (22.5%) were deemed high-risk candidates for reversal. These results are consistent with Hallam et al. [25] who reported a 47% reversal rate, with 30% of patients declining the procedure. In contrast, Tokode et al. [26] reported a higher colostomy reversal rate of 65%, although 11% of their patients also chose not to proceed with reversal.

Our study addresses a critical gap in the literature, particularly concerning UK-based practices, where data on the management of perforated diverticulitis have been limited and insufficiently explored. By focusing specifically on patients with perforated diverticulitis, we provide more targeted and local data that offer valuable insights for clinical decision-making. Our findings contribute to the growing body of evidence supporting the viability of more conservative approaches to managing complicated diverticulitis.

It is important to acknowledge the limitations of our study. First, it is a retrospective analysis from a single centre in the UK, which may limit the generalisability of our findings. Additionally, our reliance on the International Classification of Diseases (ICD) coding for case identification could have resulted in some cases being missed or misclassified. Variability in clinical practices across different institutions, each with differing levels of facilities and resources, could also affect outcomes and limit the comparability of our results to those from other settings, such as tertiary centres. Lastly, the relatively small sample size of our study may reduce its statistical power. A larger, multicentre prospective study would likely provide more robust data and offer a more comprehensive understanding of the management of complicated diverticulitis.

## Conclusions

We observed a shift towards more conservative, non-operative management of acute complicated diverticulitis with perforation over the six-year period, likely influenced by advancements in diagnostic and interventional radiology, antibiotic therapy, and minimally invasive techniques. Our data also stress that cases of perforated diverticulitis with distant extraluminal air or larger abscesses are less suited to conservative treatment, often necessitating traditional surgical interventions. Long-term follow-up showed a moderate rate of readmissions after non-resectional management, and while stoma reversal was successful in a proportion of patients, many either opted to live with the stoma or were deemed unsuitable for reversal. A larger, multicentre prospective study would likely provide more robust data on this subject.

## References

[1] Morris AM, Regenbogen SE, Hardiman KM, Hendren S (2014). Sigmoid diverticulitis: a systematic review. JAMA.

[2] Regenbogen SE, Hardiman KM, Hendren S, Morris AM (2014). Surgery for diverticulitis in the 21st century: a systematic review. JAMA Surg.

[3] Vermeulen J, Lange JF (2010). Treatment of perforated diverticulitis with generalized peritonitis: past, present, and future. World J Surg.

[4] Shaikh S, Krukowski ZH (2007). Outcome of a conservative policy for managing acute sigmoid diverticulitis. Br J Surg.

[5] Maggard MA, Zingmond D, O'Connell JB, Ko CY (2004). What proportion of patients with an ostomy (for diverticulitis) get reversed?. Am Surg.

[6] Vermeulen J, Coene PP, Van Hout NM (2009). Restoration of bowel continuity after surgery for acute perforated diverticulitis: should Hartmann's procedure be considered a one-stage procedure?. Colorectal Dis.

[7] Banerjee S, Leather AJ, Rennie JA, Samano N, Gonzalez JG, Papagrigoriadis S (2005). Feasibility and morbidity of reversal of Hartmann's. Colorectal Dis.

[8] McGillicuddy EA, Schuster KM, Davis KA, Longo WE (2009). Factors predicting morbidity and mortality in emergency colorectal procedures in elderly patients. Arch Surg.

[9] Lohrmann C, Ghanem N, Pache G, Makowiec F, Kotter E, Langer M (2005). CT in acute perforated sigmoid diverticulitis. Eur J Radiol.

[10] Ambrosetti P, Jenny A, Becker C, Terrier TF, Morel P (2000). Acute left colonic diverticulitis--compared performance of computed tomography and water-soluble contrast enema: prospective evaluation of 420 patients. Dis Colon Rectum.

[11] Kaiser AM, Jiang JK, Lake JP (2005). The management of complicated diverticulitis and the role of computed tomography. Am J Gastroenterol.

[12] Costi R, Cauchy F, Le Bian A, Honart JF, Creuze N, Smadja C (2012). Challenging a classic myth: pneumoperitoneum associated with acute diverticulitis is not an indication for open or laparoscopic emergency surgery in hemodynamically stable patients. A 10-year experience with a nonoperative treatment. Surg Endosc.

[13] Dharmarajan S, Hunt SR, Birnbaum EH, Fleshman JW, Mutch MG (2011). The efficacy of nonoperative management of acute complicated diverticulitis. Dis Colon Rectum.

[14] Sallinen VJ, Mentula PJ, Leppäniemi AK (2014). Nonoperative management of perforated diverticulitis with extraluminal air is safe and effective in selected patients. Dis Colon Rectum.

[15] Myers E, Hurley M, O'Sullivan GC, Kavanagh D, Wilson I, Winter DC (2008). Laparoscopic peritoneal lavage for generalized peritonitis due to perforated diverticulitis. Br J Surg.

[16] Alamili M, Gögenur I, Rosenberg J (2009). Acute complicated diverticulitis managed by laparoscopic lavage. Dis Colon Rectum.

[17] Karoui M, Champault A, Pautrat K, Valleur P, Cherqui D, Champault G (2009). Laparoscopic peritoneal lavage or primary anastomosis with defunctioning stoma for Hinchey 3 complicated diverticulitis: results of a comparative study. Dis Colon Rectum.

[18] Isswiasi S, El-Zahab S, Drye E (2021). The conservative management of perforated diverticulitis based on abscess size and presence of distant air. Br J Surg.

[19] World Health Organization (2024). ICD-10 International Statistical Classification of Diseases and Related Health Problems, 10th Revision, Volume 2 Instruction Manual, Fifth Edition. ICD-10 International Statistical Classification of Diseases and Related Health Problems, 10th Revision, Volume 2 Instruction Manual, Fifth Edition.

[20] Abbas S (2007). Resection and primary anastomosis in acute complicated diverticulitis, a systematic review of the literature. Int J Colorectal Dis.

[21] Mäkelä J, Kiviniemi H, Laitinen S (2002). Prevalence of perforated sigmoid diverticulitis is increasing. Dis Colon Rectum.

[22] Anaya DA, Flum DR (2005). Risk of emergency colectomy and colostomy in patients with diverticular disease. Arch Surg.

[23] Broderick-Villa G, Burchette RJ, Collins JC, Abbas MA, Haigh PI (2005). Hospitalization for acute diverticulitis does not mandate routine elective colectomy. Arch Surg.

[24] Trenti L, Kreisler E, Galvez A, Golda T, Frago R, Biondo S (2015). Long-term evolution of acute colonic diverticulitis after successful medical treatment. World J Surg.

[25] Hallam S, Mothe BS, Tirumulaju R (2018). Hartmann's procedure, reversal and rate of stoma-free survival. Ann R Coll Surg Engl.

[26] Tokode OM, Akingboye A, Coker O (2011). Factors affecting reversal following Hartmann's procedure: experience from two district general hospitals in the UK. Surg Today.

